# Effects of hyperthyroidism in the development of the appendicular skeleton and muscles of zebrafish, with notes on evolutionary developmental pathology (Evo-Devo-Path)

**DOI:** 10.1038/s41598-019-41912-9

**Published:** 2019-04-01

**Authors:** Fedor Shkil, Natalia Siomava, Elena Voronezhskaya, Rui Diogo

**Affiliations:** 1 Koltzov Institute of Developmental Biology, Russian Academy of Sciences, ul. Vavilova 26, Moscow, 119334 Russia; 2 Severtsov Institute of Ecology and Evolution, Russian Academy of Sciences, pr. Leninskii 33, Moscow, 119071 Russia; 3 Department of Anatomy, Howard University College of Medicine, 520W Street NW, 20059 Washington, DC USA

## Abstract

The hypothalamus-pituitary-thyroid (HPT) axis plays a crucial role in the metabolism, homeostasis, somatic growth and development of teleostean fishes. Thyroid hormones regulate essential biological functions such as growth and development, regulation of stress, energy expenditure, tissue compound, and psychological processes. Teleost thyroid follicles produce the same thyroid hormones as in other vertebrates: thyroxin (T4) and triiodothyronine (T3), making the zebrafish a very useful model to study hypo- and hyperthyroidism in other vertebrate taxa, including humans. Here we investigate morphological changes in T3 hyperthyroid cases in the zebrafish to better understand malformations provoked by alterations of T3 levels. In particular, we describe musculoskeletal abnormalities during the development of the zebrafish appendicular skeleton and muscles, compare our observations with those recently done by us on the normal developmental of the zebrafish, and discuss these comparisons within the context of evolutionary developmental pathology (Evo-Devo-Path), including human pathologies.

## Introduction

Thyroid hormones regulate essential biological functions such as growth and development, regulation of stress, energy expenditure, tissue compound, and multiple other processes^1–3^. They also regulate embryogenesis and control metamorphosis and molting in various animals^1,4–6^. Thyroid tissues of different shapes and forms are found in all classes of vertebrates^7^. The composition and functioning of the hypothalamus-pituitary-thyroid (HPT) axis in bony fishes are similar to those of other vertebrates^8^, except concerning the different morphology of the thyroid gland. The HPT axis plays a crucial role in the metabolism, homeostasis, somatic growth and development of teleosts. It influences the activity of a wider variety of tissues and processes than do any other endocrine axes^9,10^.

In the majority of teleosts, thyroid follicles of variable shape and size are distributed in the pharyngeal region, dispersed along the afferent artery^11^; in many species thyroid follicles are present in the head of kidneys^10,12–16^. Despite anatomical differences, teleost thyroid follicles produce the same thyroid hormones as other vertebrates: thyroxin (T4) and triiodothyronine (T3). Thyroxine (T4) has only a few direct actions and is mainly a precursor (prohormone) for triiodothyronine (T3). In turn, T3 is an active form of thyroid hormones^17^ and its predominant action is the control of complex hierarchical cascade of target genes via binding to specific receptors, ligand-activated transcription factors belonging to the nuclear receptor superfamily. T3 acts as a direct activator of expression of many genes. Binding to the receptors, T3 changes their conformation and abolish repression of gene transcription^18–21^. T3 is involved in pleiotropic processes such as osmoregulation, metabolism, growth, embryonic and postnatal development, including larva-juvenile transition and/or metamorphosis^9,10,22,23^. Thyroid hormone-dependent gene expression is known to involve a wide range of genes associated with skeletal muscle development^1^, development of eyes^24^, neural system^25,26^, and other developmental events^19^. Many metabolic and developmental processes regulated by thyroid hormones share certain similarities between fish and other animals, including primates and *Homo sapiens* in particular. In humans, thyroid hormones are also necessary for the overall physical and mental health of individuals^25,26^.

For a long time, the HPT axis was less studied in zebrafish (*Danio rerio*: Cyprinidae, Teleostei) than in other fish with a pronounced metamorphosis. In the last decades, however, zebrafish became one of the most used models in developmental biology and biomedicine, including investigations of endocrine disruptions and their effects^27–29^. These investigations revealed that thyroid hormones are essential for the normal development and physiological homeostasis during the life cycle of zebrafish^30^. In eggs, the maternal THs are present^31–33^ and play a crucial role in neurogenesis^34,35^ and early craniofacial development^36^. At early larval stages, the thyroid follicles that are diffusely distributed along the aorta are originated from endodermal tissue of the subpharynx^14,37,38^. Despite the absence of aggregation in compact glands, as seen in humans and other mammals, the follicles of zebrafish are homologous to those of the thyroid glands of mammals, and synthesize thyroid hormones T4 and T3 that are similar to those of other vertebrates. The TH signaling machinery of T3 in the zebrafish is also similar to that of mammals and other vertebrates^31^. T3 affects the transcription of target genes via binding to specific receptors, TRα and TR β with high sequence homology to mammals^30,39,40^. Particularly, it affects the metabolic rate^41,42^, cardiac function^42^, development of brain and spinal cord^34,35^, development of immune system^43^, different parts of the skeleton^36,44,45^, skin and pigment patterning^46,47^, muscle physiology^48^, and orchestrates the larva-juvenile transition^22,23^.

Interestingly, despite the increased general interest on the THs effects on zebrafish development, not so much is known on the effects of hypo- and hyperthyroidism in the anatomy of the musculoskeletal system of these fishes. Here, we investigate in detail the effects of hyperthyroidism in the developmental of the appendicular skeleton and muscles of zebrafish, compare our observations with those recently done by us on the normal developmental of these fishes^49^, and discuss our comparisons within the context of evolutionary developmental pathology (Evo-Devo-Path, see Discussion below), including human pathologies.

## Materials and Methods

Fertilized clutches (250–270 eggs) were obtained by natural mating wild-type (AB) zebrafishes. It should be noted that, starting from hatching, we fixed 3–5 fish for a period of 25 days. Taking into account that by day 10–11, 70% of the fish were dead (~150 individuals), we analyzed almost the entire population (N > 100) of individuals that were still alive. Embryos and larvae were kept at 28,5 °C ± 0.2 °C in a 25 L plastic aquaria on 14 h light/10 h dark cycle. 1/10 of the total volume of the tank water was renovated daily. The hyperthyroid status of zebrafish was provoked by the administration of 3,5,3′-thriiodothyronine (T3) (Sigma, USA) - an active form of thyroid hormones - into the water of the aquaria^22,50^. The concentration of T3 (1 ng/ml) was selected experimentally^44,45^. A higher dosage of T_3_ (≥1 ng/ml) often leads to the extremely high mortality of larvae, while a lower dosage of T_3_ (≤1 ng/ml) does not evoke distinguishable changes in morphology. The tank water (1/10 of total volume) was renovated daily with the administration of T3 up to a predetermined concentration. The onset of hormonal treatment was at the segmentation period of embryonic development. The duration of a hormone administration was for 25 days, until appearance of squamation in zebrafishes. The newly hatched larvae were fed on TertraMin baby (Tetra, Germany) and NovoTom artemia (JBL, Germany). A week after the foraging onset, the diet was changed to TertraMin Junior (Tetra, Germany) and Artemia Salina shrimps (Artemia Koral, Russia). Animals were euthanized at stage 48 hpf with NL 3 mm; then, the ones that were alive were euthanized every day starting from the hatching, and so on: 3 to 5 fishes were euthanized with the overdose of anesthetic MS-222 (Sigma-Aldrich, Germany) and fixed for 12–14 h at 4 °C in 4% paraformaldehyde (Panreac, Spain) in 0.01 M phosphate buffer saline (PBS pH 7.4, Gibco, Germany) supplemented with 10 μl of 0.5% Alizarin Red (Sigma, USA) diluted in water per 1 ml of the fixation solution. After fixation, samples were washed three times in PBS and stored in PBS with antimicrobial agent 0.1% Thymol (Fluka Analytical, USA) at 4 °C. The lattest fish samples were collected at stage 31dpf with maximal SL 9.2 mm.

The developmental stage of the specimens was estimated by the length of the body. The notochord length (NL) of preflexion fish larvae was measured from the anterior end of the upper jaw to posterior tip of notochord^51^. In fishes with a bending posterior tip of the notochord, the standard length (SL) was measured from the anterior end of the upper jaw to the posterior end of the hypurals^51,52^. The length of every fish was measured under the stereomicroscope Olympus SZX7 with an ocular micrometer.

After several washes in fresh PBS, samples were transferred in PBS-TX (5% Triton X-100 in PBS) and incubated at 10 °C for 72 h with three-four changes of PBS-TX. Then, samples were incubated in phalloidin-Alexa 488 (Invitrogen, Molecular Probes, A 12379) in dilution 1:500 and TOTO (TOTO™-3 Iodide, ThermoFisher Scientific, T3604) in dilution 1:1000 in PBS-TX for 24–48 h. After three washes in PBS, samples were mounted in 85% fructose in PBS (through gradual scale 30%, 50% and 70%) between two cover slips and stored at room temperature upon examination.

We performed and ontological exam of various case studies with different levels of defects. Notably, the pathological series analyzed in the present work does not reflect the ontological progress of the hyperthyroidism. In total, more than 100 specimens at different stages were examined as whole-mounts under the laser scanning microscope Leica TCS SP5 (Leica, Germany) equipped with the Ar 488 laser for the phalloidin visualization, the DPSS 561 laser for the alizarin visualization, and DPSS 633 laser for nuclei visualization. The laser intensity and wavelength-filter configuration were set up the capture all details. When necessary, fish larvae were scanned from both sides. For each larva, 150–250 optical sections with thickness 1.0–1.5 μm were taken and processed with Leica LAS AF (Leica, Germany) and inspected with the ImageJ (NIH, USA) software. Series of optical sections containing relevant structures were projected into a single image and exported as a TIFF file. Due to a variant number of optical sections required for every image, the brightness and contrast were adjusted with ImageJ for each panel separately. The nomenclature of the developing zebrafish appendicular muscles follows that of adult zebrafishes in Siomava & Diogo^53^. This latter paper is crucial for the present study, as it describes the normal development of appendicular muscles in untreated (control) zebrafishes, i.e. it provides the anatomical basis for the comparisons with the abnormal development of these muscles, reported in the present paper.

All zebrafish experiments were approved by ethics committees of the Russian Academy of Sciences. All procedures were carried out according to the guidelines and following the laws and ethics of Russian Federation and USA.

## Results

### General effects of exogenous T3 in the studied zebrafish

**Figure 1:**
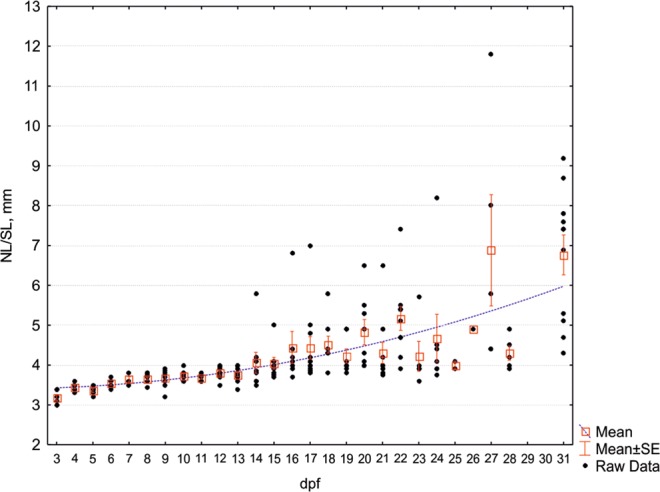
Growth dynamic of T3 treated zebrafish. 1–14 dpf notochord length (NL, mm), 15–31 dpf - standard length (SL, mm). Points - size of individual, boxes - average size per sample, whiskers - standard error, dashed line - polynomial trend, dpf - days post fertilization.

**Figure 2:**
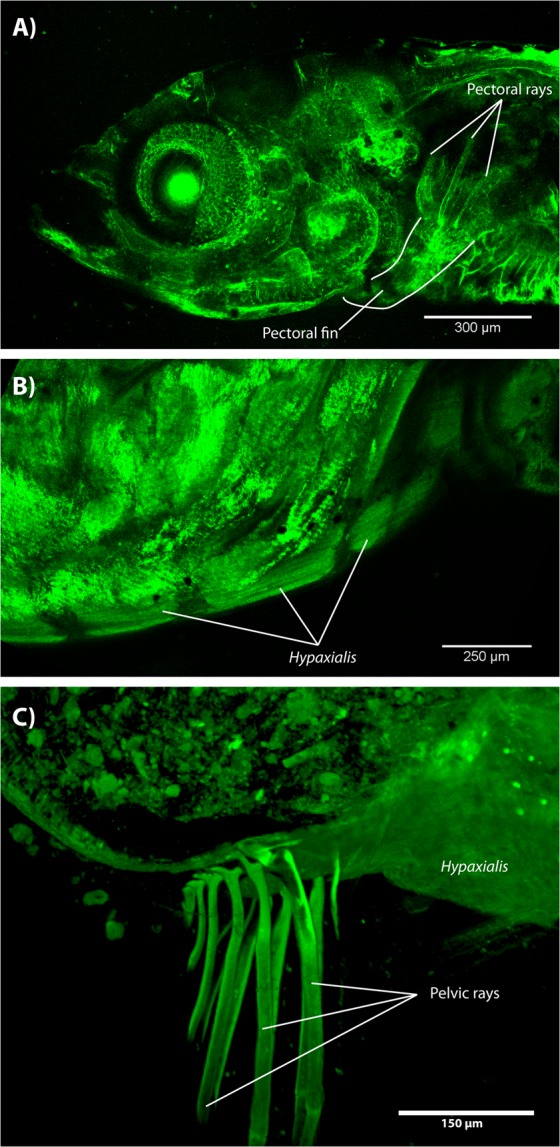
Paired fins in T3 treated zebrafish. Pectoral fin was rotated counter-clockwise (**A**) 3.8 mm SL. Pelvic fins were absent in most fish (**B**) 8.7 mm SL. A single individual had pelvic fin developed (**C**) 8.5 mm SL. In all panels, anterior is to the left, dorsal is to the top.

The effects of the T3 hormone were manifested since the very early stages of zebrafish development. The first features that distinguished the T3 treated fish were deviations in behaviour, changes in the larval pigment patterning, and lateral or dorsal curvature of the trunk. The underdevelopment of the gas bladder and numerous skeletal abnormalities resulted in deviant locomotion of the experimental fish. Additionally, experimental fish were more active and anxious, when compared to untreated fish. These behavioural deviations were likely caused by the alterations in the developing nervous system and/or metabolic levels that are known to occur in hypothyroidism conditions. The axial skeleton of specimens incubated in exogenous T3 was often deformed and bore signs of scoliosis. Many treated fish had a non-filled swim bladder. These deformities could be detected as early as the onset of active feeding, at 3.6–3.7 mm NL. Likely due to the T3 treatment, the mortality was very high and by day 10–11 more than 70% of larvae were dead. From very early stages, we could distinguish mainly two muscle phenotypes: specimens with severely underdeveloped muscles and specimens with light muscular defects, if any. Variation in size (SL) increased with age (Fig. 1). Besides variation in size, certain morphological alterations were apparent in the T3 treated zebrafish. One of those features was the positioning of the pectoral fin. The entire fin was rotated counter clockwise and the tip of the fin pointed posterodorsally. At 3.8 mm SL, when several lepidotrichia developed, they retained the general position of the fin (Fig. 2A).

Another peculiar feature was the entire absence of pelvic fins in T3 treated specimens (Fig. 2B), except in a single case: a specimen that had a pelvic appendage on one side of the body only present at 8.5 mm SL (Fig. 2C). The pelvic girdle was poorly developed and the fin itself was severely deformed: fin rays were perpendicular to the girdle and approximately 90° rotated in the direction opposite to the pectoral fin. We could not discern any muscles going to the pelvic fin rays. The protractor and retractor ischii were slightly attached to the pelvic girdle.

### Bone development and malformations

Generally, the effects of the T3 treatment on the appendicular skeletal development of the zebrafish used in the current experiment are similar to those seen in the skeleton, including the head, of the zebrafish and of African large barbs^45^. Therefore, here we briefly describe the main developmental skeletal malformations observed in the present study and focus on the muscle malformations, which were never studied in detail in the zebrafish.

**Figure 3:**
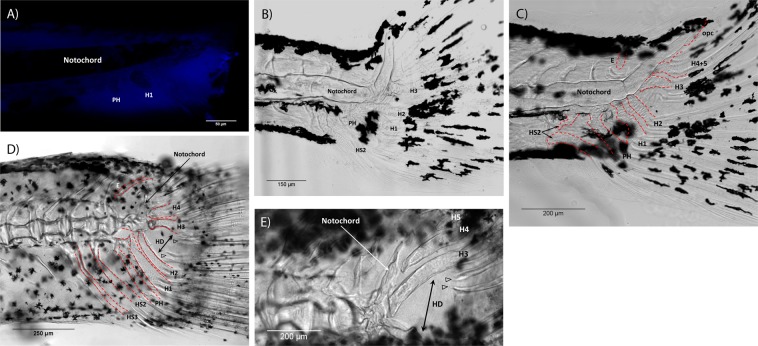
Skeleton of the caudal fin in T3 treated zebrafish. Hypural 1 (H1) and parhypural (PH) were clearly visible at 3.6 mm NL (**A**). Notochord and hypurals were deformed, resulting in scoliosis (**B**) 4.1 mm SL. Caudal fin of fish 4.9 mm SL (**C**) hypurals were irregularly shaped; hypurals 4 and 5 were fused (H4 + 5); haemal spine was broken (HS2, arrows); epural (**E**) was present. Lobes of the caudal fin were of a similar size with a widen hypural diastema (HD) (**D**) 7.8 mm SL. The very tip of the notochord was heavily bent and atrophied (**E**) 9.2 mm SL. Red dotted lines in panels C and D outline the named caudal bones. In all panels, anterior is to the left, dorsal is to the top.

The early development of hypurals in the treated zebrafish was similar to that seen in the normal phenotype. The first hypaxial elements to develop were the parhypural (PH) and the hypural 1 (H1), which were first noticed at 3.5 mm NL (12 dpf) and became clearly visible at 3.6 mm NL (11 dpf) (Fig. 3A). At 3.8 mm NL, the hypural complex of treated fish looked normally shaped. Starting from 4.0 mm SL, we observed various bone deformations. The hypurals acquired irregular shapes and the caudal vertebra became curved resulting into different degrees of scoliosis (Fig. 3B). The notochord was often hogged 90° upward at the junction of the ural bones U1 and U2+, where the hypural diastema is located. The degree of deformations increased with age. At 4.9 mm SL, most individuals had severely altered shapes of the caudal bones. The hypurals H4 and H5 were often (but not always) fused and the hypural diastema (HD) was widened (Fig. 3C). In some cases, the haemal spine of pleural 2 (HS2) was completely detached from the notochord and lied freely within the musculature (Fig. 3C, black arrows). From 7 mm SL onward, the notochord was heavily bent (Fig. 3D) and often ossified abnormally (Fig. 3E). Notably, the hypurals H3-H5 might look normal in the same individuals. Overall, the caudal fin appears to be bifurcated with similarly sized dorsal and ventral parts and a wide HD between them (Fig. 3C,D). Some middle rays lost their connection to hypural bones.

**Figure 4:**
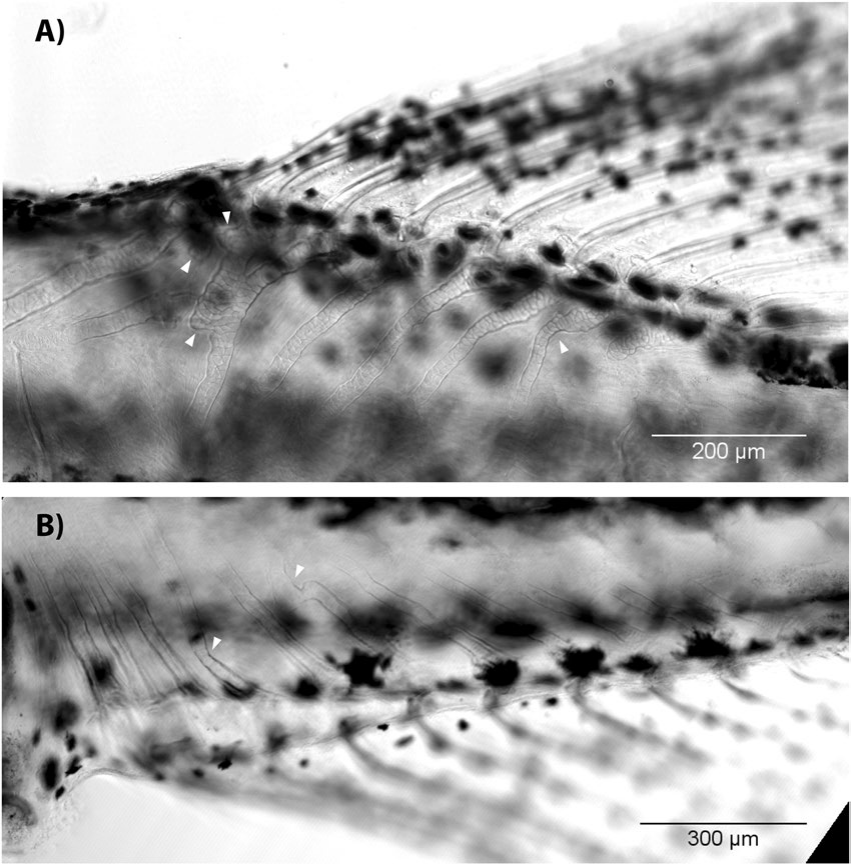
Skeleton of the dorsal and anal fins in T3 treated zebrafish. Radials of the dorsal (**A**, 6.9 mm SL) and anal (**B**, 7.0 mm SL) fins are often deformed and fused (white arrow heads). In both panels, anterior is to the left, dorsal is to the top.

We did not observe defects of the dorsal and anal fin skeleton during early development of the treated fishes. Individuals at 5.1 mm SL and older, often had deformations and fusions of radials in both the dorsal (Fig. 4A) and the anal (Fig. 4B) fins. The first unbranched ray was sometimes absent in either the dorsal or the anal, or in both fins. The total number of rays was sometimes reduced: some individual had 11 anal rays fin instead of 13.

The larval pectoral fin and girdle developed without apparent defects. However, after the precocious transition to the adult state accompanied by the appearance of fin rays, numerous abnormalities in shape and the proportions of the proximal radials were observed. Similar malformations were previously described for zebrafish and barbs (see^45^ for more details).

### Muscle development and malformations

**Figure 5:**
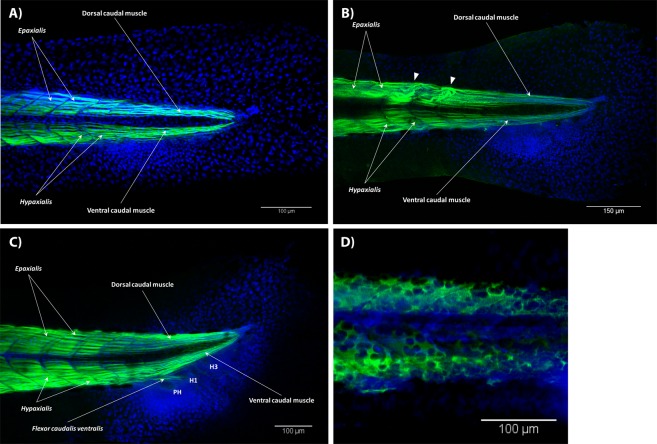
Early development of the caudal fin musculature in T3 treated zebrafish. A general view of the caudal fin and a condensation of cells on its ventral side at 3.4 mm NL (**A**). Curly muscle fibers in the epaxialis in a fish at 3.6 mm NL (**B**). Development of the intrinsic caudal muscles started with an outgrowth of the flexor caudalis ventralis and is shown in a fish at 3.8 mm NL (**C**). Enlarged nuclei in the muscular tissue in a fish at 4.0 mm NL (**D**). In all panels, anterior is to the left, dorsal is to the top.

**Figure 6:**
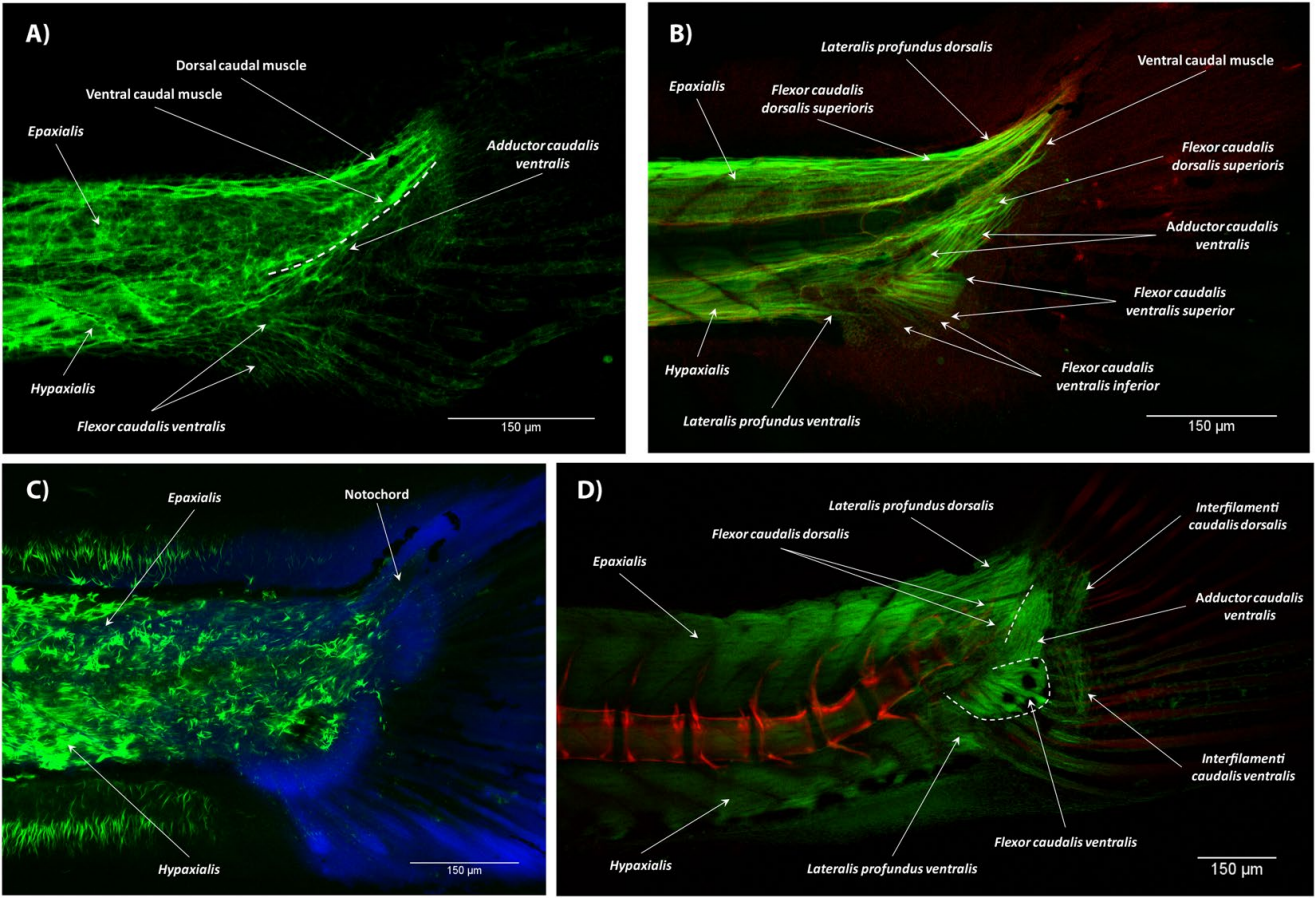
Late development of the caudal fin musculature in T3 treated zebrafish. Underdeveloped caudal muscles form in one fish 4.0 mm SL (**A**) and almost normally developed in another fish 4.0 mm SL (**B**). A net of disorganized muscle fibers in the caudal fin and abnormal muscle development in the dorsal and anal fins in a fish 4.4 mm SL (**C**). The adult-like muscle configuration of the caudal fin is achieved by the development of the interfilamenti caudalis dorsalis and ventralis (**D**) 5.0 SL. In all panels, anterior is to the left, dorsal is to the top.

At 3.4 mm NL, we observed a condensation of cells on the ventral side of the tail, where muscles and bones will later develop (Fig. 5A). The first clear muscle defects seen in the T3 treated fishes were detected in specimens at 3.6 mm NL. Muscle distortions and curly muscle fibers were present in several posterior (caudal) myomeres (Fig. 5B). The first fibers of the caudal muscles started growing on the ventral side at 3.7 mm NL. They grew quickly and were clearly seen at 3.8 mm NL (Fig. 5C). Starting from 3.9 mm NL, we could distinguish two phenotypes. Specimens showing the first phenotype had a significantly reduced amount of muscle fibers that possessed enlarged nuclei (Fig. 5D). An example of such phenotype is shown in Fig. 6A. The other group of zebrafish had only minor deformations in the amount and overall shape of muscles (Fig. 6B). Apparently, specimen from the first group had major functional problems and their mass death (approximately 70%) at 10–11 days postfertilization provided an observed surge of a mortality rate. The mortality rate at stage 4 mm SL was increased to approximately 80%. Only specimens with a low sensitivity to the hormone, and therefore with mild deformations, survived the treatment, and were analyzed at later stages of development. In specimens with mild defects, we identified most of the intrinsic caudal muscles at 4.0 mm SL, including the flexor caudalis ventralis superior and inferior, flexor caudalis dorsalis superior and inferior, adductor caudalis ventralis, lateralis caudalis dorsalis and ventralis, as well as some fibers of the ventral caudal muscle (Fig. 6B). In some cases, no muscles could be distinguished within a continuous net of disorganized muscle fibers (Fig. 6C). By 4.5 mm SL most fishes with severe defects were eliminated from the population and only those with light defects survived. At 4.9 mm SL, the interradialis caudalis was developed and the adult-like configuration of muscles was achieved at 5.0 mm SL by the appearance of the interfilamenti caudalis dorsalis and ventralis (Fig. 6D). At this stage, the development of the caudal fin musculature was complete. Similar muscle distortions, i.e. curly fibers, fibers organized in nets, short disorganized fibers, large nuclei and reduction in number, were also present in the superficial layer of the caudal musculature.

**Figure 7:**
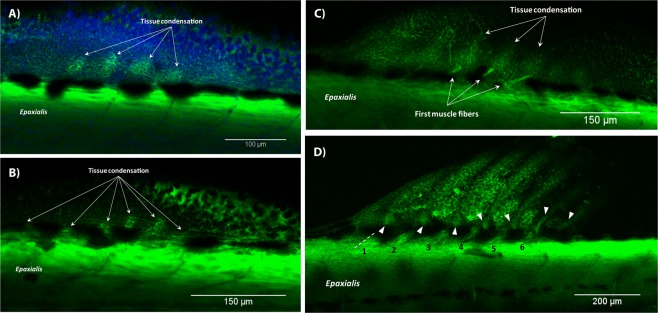
Development of the dorsal fin in T3 treated zebrafish. Condensation of mesenchyme in the dorsal fin at 4.0 SL (**A**). At 4.2 mm SL, stripes of the mesenchymal condensation were slightly distorted (**B**). Units of muscle fibers could be wrongly inclined (**C**) 4.2 SL. The number of the muscular units did not always correspond to the number of rays (**D**). White arrow heads show bases of seven dorsal rays in a fish 5.5 mm SL, numbers 1–6 correspond to the muscular units. In all panels, anterior is to the left, dorsal is to the top.

**Figure 8:**
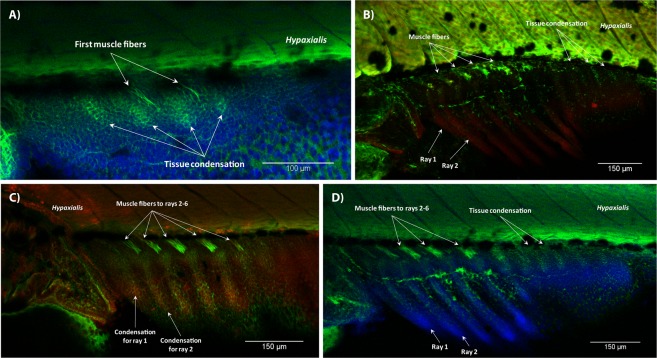
Development of the anal fin in T3 treated zebrafish. Mesenchymal condensations and first muscle fibers organized in serially repeated units (**A**); 4.0 SL. Strong (**B**) and weak (**C**) effects of T3 hormone in specimens 4.2 mm SL. Some individuals were more resistant to the hormone and developed normally (**D**); 4.9 mm SL. In all panels, anterior is to the left, dorsal is to the top.

The development of the dorsal and anal fins was initiated at 3.8 mm SL. Chonrocytes of prospective radials were organized in multiple stripes at 3.9 mm SL (Fig. 7A). At 4.0 mm SL, the first muscle fibers developed in the anal fin of some specimens, while the dorsal fin retained the mesenchymal condensations (Figs 7A and 8A). Starting from 4.2 mm SL, we could distinguish phenotypes with mild and severe muscle deformations. These phenotypes, however, did not necessarily have a one-to-one correspondence to the light/severe muscle phenotypes described above. In the dorsal fin, cartilage proximal radials became slightly irregular in shape (Fig. 7B) resulting into muscle fibers to be unevenly inclined (Fig. 7C). In the anal fin, muscles were also slightly disorganized. In some 4.2 mm SL individuals, muscles of the anal fin formed a single layer attached to slightly bent lepidotrichia (Fig. 8B). In other individuals of the same length, muscles were more developed (Fig. 8C) and several units consisted of two muscle layers. Development of lepidotrichia was not yet accomplished in fish at 4.2 mm SL (Fig. 8C). All three muscles – the erector, depressor, and inclinator – were present in at least one muscular unit of the anal fin in specimens starting from 5.0 mm SL. In some fish, the number of the muscular units did not correspond to the total number of rays. For example, the last dorsal ray of the fish shown in Fig. 7D lacked muscles. In some individuals at 4.3 mm SL and older, we observed an excessive muscle development in the dorsal and anal fin folds. In this case, muscle fibers were not organized in units and did not seem to be attached to any kind of bony structures (Fig. 6C). In contrast to the muscle malformations in the anal and dorsal fins described above, many zebrafish developed normally (Fig. 8D).

**Figure 9:**
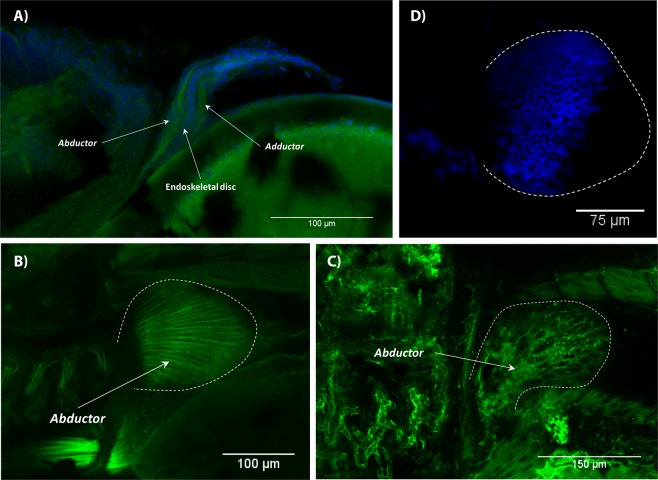
Early development of the pectoral fin in T3 treated zebrafish. Two muscle layers were present in the pectoral fin at 3.2 mm NL (**A**). Strong (**B**; 3.3 mm NL) and weak (**C**; 3.5 mm NL) effects of T3 hormone on pectoral muscles. No apparent defects were observed in the endoskeletal disc (**D**) 3.6 mm NL. In all panels, anterior is to the left. Panel A: left side of the body to the top. Panels (C–E) dorsal is to the top.

**Figure 10:**
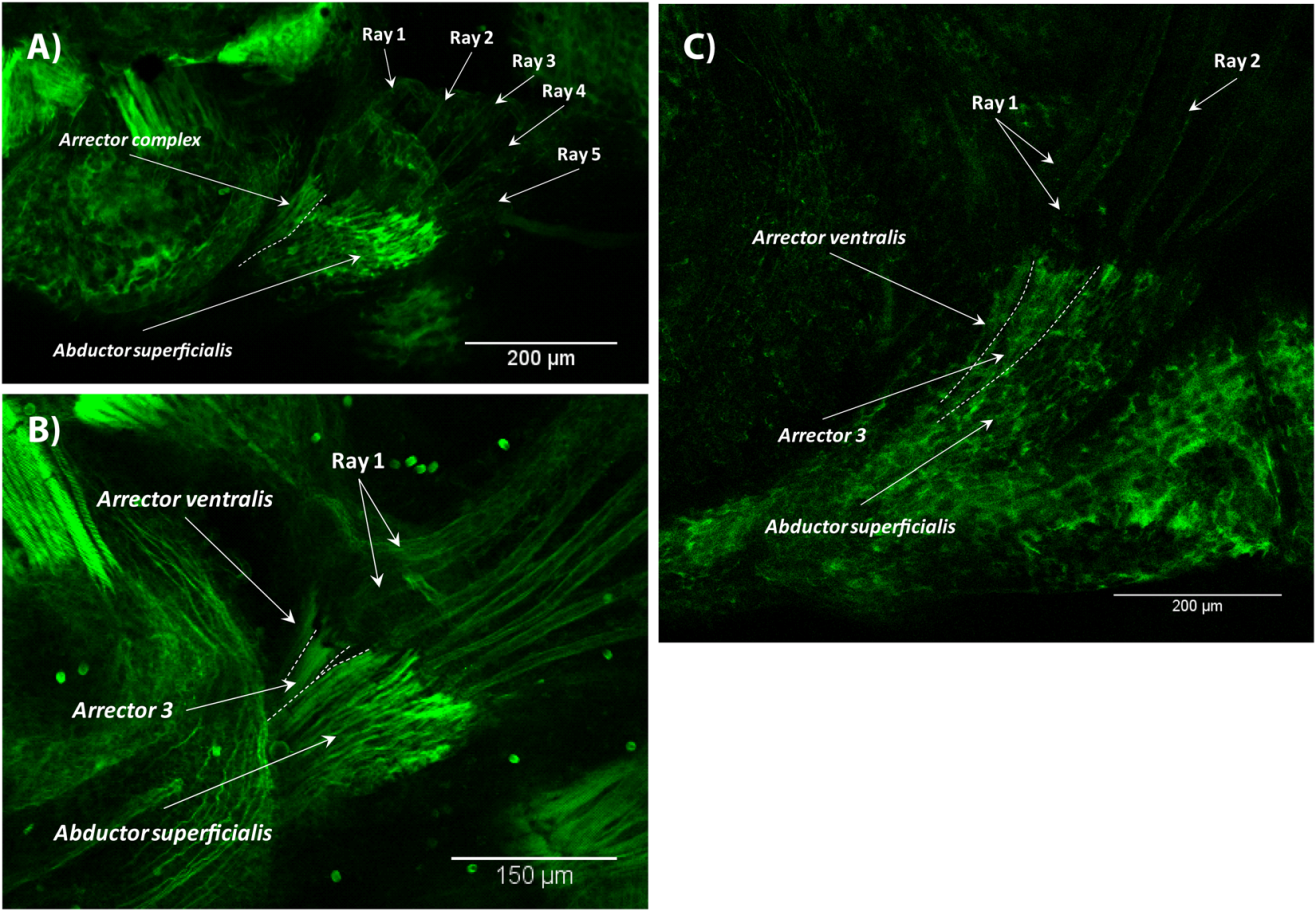
Late development of the pectoral fin in T3 treated zebrafish. The arrector ventralis complex was seen as early as 3.9 mm SL (**A**). By 4.0 mm SL, it was subdivided into the arrector ventralis and arrector-3 (**B**). In some specimens with the adult state of the pectoral musculature, muscles were slightly underdeveloped and disorganized (**C**) 4.9 mm SL. In all panels, anterior is to the left, dorsal is to the top.

In the larval pectoral fin, the endoskeletal disk as well as adductor and abductor muscle masses were already present at 3.2 mm NL (Fig. 9A). At 3.3–3.5 mm NL, muscles were straight without evident deformation in some specimens. They extended all the way to the edge of the disk (Fig. 9B). In other specimens, muscle fibers were disorganized and underdeveloped. They formed a net (Fig. 9C) similar to that in the caudal fin (Fig. 6A). No remarkable defects of the endoskeletal disc were observed in these specimens (Fig. 9D). Apparently, zebrafish with heavily underdeveloped muscles could not survive and died. Within those that were more resistant to T3 and that survived the treatment, the arrector complex developed on the ventral side of the pectoral fin as early as 3.9 mm SL (Fig. 10A); at 4.0 mm SL, it was already subdivided into the arrector ventralis and arrector-3 (Fig. 10B). Within some of these more resistant fishes, muscles were slightly disorganized and less dense but all pectoral muscles were present (Fig. 10C).

As noted above, only one T3 treated zebrafish had a pelvic appendage at 8.5 mm SL, on one side of the body only (Fig. 2C). We could not discern any muscles going to the pelvic fin rays, while the protractor and retractor ischii were slightly attached to the pelvic girdle.

## Discussion

### Accelerated development and differentiation

Effects of the exogenous T3 in the treated zebrafish were detected in fry starting from very early stages. Unlike some previous studies^22^, we observed external changes in fish younger than 5 mm (3 weeks post fertilization). An unusual position of the pectoral fin was already apparent in specimens <4 mm SL. Starting from 4.0 mm SL, we observed various deformations in the caudal skeleton, including various degree of scoliosis. They were also noted by Brown (1997) in the zebrafish and^11^ in flatfishes. It is likely that such malformations resulted from the accelerated morphogenesis of skeletal structures and their early ossification, which was also observed in our experiments. Similar developmental effects and malformations were reported for non-muscular systems in zebrafish and African barbs in previous investigations^45^. In fact, accelerated development and differentiation are known effects of thyroid hormones in general. They were confirmed, for instance, in various fish species^6,22,54–56^, axolotls^22^, and reptiles^4^. In our experimental zebrafish, we recorded development of hypural elements starting at 3.5–3.6 mm NL, which falls within the normal time variation^57,58^. Further development of the hypural complex proceeds faster and is often complete by 4.1 mm NL. Within normal development, the last hypural (H5) has been reported to develop at 5.3 mm TL^59^ or at 5.0 mm NL^60^.

The same trend of acceleration was also present in the development of the musculature. The first muscle fibers growing on the ventral side of the caudal fin were observed as early as 3.7 mm NL; these muscle fibers became clearly visible at 3.8 mm NL. In normal development, the outgrowth of the flexor caudalis ventralis and aductor caudalis ventralis happens usually at 4.4 mm SL^61^. By 4.0 mm SL, most of the intrinsic caudal muscles were already formed, as noted above (Fig. 6B), while this only occurs at 5.5 mm SL, usually within normal development^61^. At 4.9 mm SL, T3 treated specimens possessed an interradialis caudalis, which usually only appears at 6.4 mm SL in normal development; the interfilamenti caudalis dorsalis and ventralis were present at 5.0 mm SL in T3 treated fish (Fig. 6D), while in the normal phenotype they appear at 6.7 mm SL^61^.

Regarding the anal fin, the first muscle fibers were observed in treated fishes at 4.0 mm SL (Fig. 8A), while in normal development they appear at 5.8 mm SL^61^. Moreover, the earliest stage when all three muscles of a unit (the erector, depressor, and inclinator) were formed in at least one muscular unit was 5.0 mm SL, while in normal development it is usually at 6.4 mm SL^61^.

Development of the pectoral fin was also accelerated in T3 treated fish. The arrector complex was already subdivided into the arrector ventralis and arrector-3 at 4.0 mm SL (Fig. 10B): this only usually happens at 6.7 mm SL in normal development^61^. Accelerated maturation of non-muscular structures of the pectoral fin was also noted by other authors in the zebrafish^22^ and the salmon species *Oncorhynchus keta*^54^. Another defect in pectoral fin development is the alteration of its orientation during maturation of the zebrafish. During normal development, larval pectoral fins have been reported to orient vertically with reference to the anteroposterior body axis during the first two weeks of a fry’s life and to gradually rotate into a near horizontal position during the third week (5.4–5.8 mm), marking the transition to the adult stage^62^. While we observe a maturation of the pectoral fin in terms of musculature, we did not observe a marked rotation of the fin during development of the T3 treated fish. Moreover, the pectoral fin was notably rotated counter clockwise and retained this juvenile position throughout later stages of development (Fig. 2A).

Concurrently with the general accelerated development and differentiation of the structures discussed just above, we also noted a profound effect of T3 on growth rates. Unlike the zebrafish population observed in the experiments undertaken by Brown^22^, our specimens grew smaller in size: this can be partially be explained by the curvature of the backbone and severe scoliosis in some of our treated fishes. In fact, the effect of thyroid hormones on growth remains unclear. T3 is known to act bimodally, i.e. it can enhance anabolic or catabolic metabolisms depending on the dosage^63^. Growth retardations have been reported to occur in different salmon species^54,64^ and brown trouts^65^, but numerous authors reported acceleration of growth in various fish and reptiles, e.g. tilapia^66^, carp^67^, milkfish^63^, dwarf gouramy^68^ and striped bass^69^, python^70^, and also cessation of molting in snakes^71^. Along with these apparently contradictory results, some authors reported no effect of thyroid hormones in growth and in some other fundamental developmental processes in fish, e.g. in guppy^72^.

### Consequences of hyperthyroidism

A great number of parameters determine the effects of thyroid hormones. The age, gender, nutrition, health conditions, physiological state of the animal, and the diet^2^ as well as captivity^73^ and numerous environmental factors have been shown to influence the activity of the HPT axis, levels of endogenous thyroid hormones, activity of deiodinases, and accessibility of TRs and, therefore, the overall response to thyroid treatments. In poikiloterm animals such as fish, amphibians and reptiles rearing temperatures can alter the metabolic response to thyroid hormones^7,74–80^. Diurnal and circadian rhythms influence the action of thyroid hormones in fish^81^. Seasonal fluctuations of HPT axis activity, found in snakes, lizards, and turtles^7,73,82^, may also alter the action. All these factors and many others lead to conflicting reports from different laboratories. The effects of thyroid hormone administration are often difficult to compare^63^ and manifestation and the final outcome of hyperthyroidism can vary between animals.

In fact, it should be noted that the action of thyroid hormones is largely pleiotropic. A large number of anabolic and catabolic genes can respond to the T3 treatment and contribute to effects on various systems of the organism and its metabolism in general^19,24^. The complexity of the response is also determined by the dose and the nature (synthetic or organic, T3 or T4) of the hormone. These particular qualities make it difficult, if at all possible, to distinguish between different degrees of hyperthyroidism and thyrotoxic state. Thus, all genes involved in a direct interaction with the T3 molecule have their expression consequently altered via other possible pathways (e.g. cortisol, growth hormone, melatonin, and various stress hormones). It has also been shown that certain regulatory regions in the genome can be dramatically remodeled by T3 and, therefore, the expression of neighboring non-T3-regulated genes can also be altered^19^. Recent studies report more than 10 transcriptional factors (and therefore all their downstream genes) to be differentially expressed in fish supplemented with exogenous T3^24^. Almost one hundred genes affected by the hormone are involved in the development of the pectoral fin and 48 genes are involved in development of the notochord^24^. Variation in the sensitivity to thyroid hormones can also be determined by genetic properties of the organism, e.g. a number of mutations have been shown to provide a resistance^31^. Below, we will therefore try to outline some effects that seem to be shared among different species by taking also into account our own observations.

Hyperthyroidism and thyrotoxicosis have been shown to induce weight loss throughout the course of life in humans^83–86^ and other animals^22,87–89^. In humans, hyperthyroidism during neonatal period can also lead to the growth retardation^90^. Excess of thyroid hormones in childhood and the juvenile period often leads to the accelerated skeletal development and rapid growth, but the advanced bone age results into the early cessation of growth^91^. Patients with such characteristics have a persistent short stature^91,92^. Untreated hypothyroidism in childhood can lead to the growth arrest and an increased risk of fractures^92^. Importantly, similar effects of thyroid hormones were observed in fish treated with an excessive amount of T3 during our experiments. Accelerated development of skeletal elements resulted in numerous deformations, including scoliosis, fusions of hypural and radial cartilages/bones, and fractures of caudal elements (Figs 3 and 4).

Another prominent symptom of hyperthyroidism in humans is muscular atrophy resulting into the weakness of proximal muscles, loss of muscle mass and subsequent sarcopenia^84,86,93^. The myofibrillar degradation observed in the T3 treated zebrafish studied by us strongly supports the view that myofibrillar content of muscle is often decreased in hyperthyroidism^93^. Within other fishes, in the Japanese flounder *Purdichthys olivaceus*, it has been shown that thyroid hormones are involved in the transition of muscle proteins during metamorphosis^1^. In rats, thyroid hormones regulate fetal to adult transition of cardiac myosin^94^. These facts suggest that the muscle fiber atrophy that occurred in our T3 treated fish could potentially result from an incorrect reorganization of the larval to adult metabolism. The engagement of thyroid hormones in both anabolic and catabolic pathways also suggests that perturbations in the balance between these two processes can stimulate excessive muscle growth such as the one observed in the dorsal and anal fin folds in our T3 treated zebrafish (Fig. 6C; see above).

Importantly, in spite of numerous reports on myopathy, changes in muscle proteins and fiber content, there are no reports of alteration in the topology and specific attachment sites of muscles in human hyperthyroidism^84,86,93^. Our results in fish conform to these observations. Even such drastic changes as the occurrence of an almost complete bifurcation of the caudal fin into dorsal and ventral lobes and/or the atrophy of the tip of the notochord did not alter the specific attachment of muscles, when they were present. This parallels between the pathological development in zebrafish and humans, two clades that are phylogenetically very distant, further support the idea that is the basis of the new sub-field of Evo-Devo that has been developed by us and other researchers recently: Evo-Devo-Path, or Evolutionary Developmental Pathology (see e.g.^95–98^). That is, these recent studies have shown that even very distant lineages share similar developmental, evolutionary and developmental patterns, because of the highly constrained character of biological evolution. these recent studies have also stressed that the vast majority of the works on the links between evolution, development and pathology have, unfortunately, focused mainly on osteological or superficial features (e.g., absence of a certain bone, shape of head), with almost no information been available about the muscular system of non-human animals with severe malformations. The present paper is precisely part of an ongoing effort to change this status quo. In particular, it is hoped that the data obtained can be used in future research about, and help in understanding, human hyperthyroidism, by being one of the first detailed studies on how muscle anatomy is affected in the abnormal development of hyperthyroidism. It is therefore also hoped that this paper will further stimulate the development of Evo-Devo-Path, and in particular of myological studies that will contribute to link fields such as comparative anatomy, zoology, evolutionary developmental biology, developmental biology, pathology, and medicine in general.

## Data Availability

All scans generated and analyzed during the current study are available from the corresponding author upon reasonable request.
